# Electrospun Lignin/ZnO Nanofibrous Membranes for Self‐Powered Ultrasensitive Flexible Airflow Sensor and Wearable Device

**DOI:** 10.1002/adma.202502211

**Published:** 2025-07-02

**Authors:** Yifei Zhan, Jade Poisson, Xintong Meng, Zengbin Wang, Lizhen Chen, Tun‐Hui Wu, Robert Koehler, Kai Zhang

**Affiliations:** ^1^ Sustainable Materials and Chemistry Department of Wood Technology and Wood‐Based Composites University of Göttingen 37077 Göttingen Germany; ^2^ Faculty of Engineering and Health University of Applied Sciences and Arts Von‐Ossietzky‐Straße 99 37085 Göttingen Germany; ^3^ Fraunhofer Institute for Surface Engineering and Thin Films IST Application Center for Plasma and Photonics Von‐Ossietzky‐Straße, 99 37085 Göttingen Germany; ^4^ Wöhler Research Institute for Sustainable Chemistry (WISCh) University of Göttingen Tammannstr. 2 D‐37077 Göttingen Germany

**Keywords:** airflow sensors, electrospinning, flexible electronics, wearable devices

## Abstract

The interest and demand for flexible sensors and wearable devices are rapidly growing. The added benefit of electricity generation, enabling gas sensors to be self‐powered, increases the applicability of these devices for flexible and wearable airflow sensors. Inspired by water evaporation‐induced power generation, this study explores its potential in sensing applications, which has not yet been explored in detail. Electrospinning technology is used to prepare superhydrophilic lignin/ZnO nanofibrous membranes with a ZnO nanoparticle layer, capable of generating at least 100 mV (which allows it to power its own signal transduction). The membrane is highly sensitive to variations in airflow, enabling its use as an ultrasensitive and flexible airflow sensor. This sensor demonstrates exceptional performance, including a fast response time (0.65 s), broad detection range (with lower detection limit down to 0.25 and upper detection limit of 3 m s^−1^), and extremely high airflow velocity detection accuracy. Beyond these, it can serve as a wearable sensor for sweat monitoring, motion detection, and breath monitoring (to accurately detect breathing rate, intensity and variations in speech). Such self‐powered, ultrasensitive, and flexible lignin/ZnO airflow sensors provide novel potential to advance the development of smart textiles and wearable electronics.

## Graphical abstract

This study explores the novel sensing applications of water evaporation‐induced power generation, by using lignin/ZnO nanofibrous membranes for sensing airflow through output voltage variation. Obtained lignin/ZnO airflow sensors are self‐powered, precise, and quick‐responding, and can be used as wearable devices for breath monitor, surrounding movement sensor, and sweat monitor.

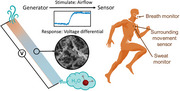

## 1 Introduction

Airflow sensors are important functional components in numerous industrial fields including environmental monitoring, biomedical engineering, aerospace, somatosensory systems and wearable devices.^[^ ^1^ ^]^ Traditional airflow sensors are mainly based on the theory of detecting the mechanical deformation of the sensing components by piezoresistive, capacitive, optical or magnetic effect,^[^ ^2^ ^]^ or the heat exchange between the heated materials (such as metallic wire and film) and the airflow by the change of the resistance.^[^ ^3^ ^]^ However, as sensing technology has advanced, the use of traditional sensors has been limited due to their rigid and cumbersome form factors.^[^ ^4^ ^]^ For instance, the flexibility of the sensor is crucial for generating higher‐quality signals and allowing for the continuous and stable monitoring of these signals without interfering with daily activities.^[^ ^5^ ^]^ Meanwhile, the dependence of traditional sensors on power sources also limits their use, primarily due to the considerable mass and volume of batteries, as well as the potential environmental pollution and health hazards associated with battery materials.^[^ ^6^ ^]^ Autonomous capabilities, which eliminates the need for external power, have increasingly become a critical factor that broadens the applicability potential of airflow sensors. Consequently, developing self‐powered flexible airflow sensors is both highly desirable and necessary.

Water evaporation‐induced generators (WEIGs) generate streaming voltage and current through the selective transport of ions across the channels within the electric double layer, driven by water flow,^[^ ^7^ ^]^ which is sustained by continuous capillary action and evaporation.^[^ ^8^ ^]^ WEIGs can harvest thermal energy from the ambient environment to generate electrical power,^[^ ^9^ ^]^ which is particularly useful in small electronic devices, capacitors, electrochemical deposition, and electrochemical cells. Additionally, higher current and voltage can be achieved by simply assembling these generators in series and parallel.^[^ ^10^ ^]^ We observed that the power generation capability of the WEIGs heavily depends on the rate of water evaporation on their surface.^[^ ^11^ ^]^ Thus, herein we report a novel method for airflow sensing, which involves detecting the power generation of a WEIG, and assessing the impact of water evaporation rate on the power generation by modulating the airflow conditions of the exposed surface.

Electrospinning represents a straightforward and highly efficient technology for the fabrication of nanofiber‐based materials.^[^ ^12^ ^]^ The nanofibrous membranes prepared by this method possess many advantages such as, high specific surface area, high porosity, adjustable structures and super flexibility,^[^ ^13^ ^]^ which make them excellent candidates for flexible devices.^[^ ^14^ ^]^ Moreover, the high porosity and specific surface area of nanofibrous membranes can provide channels for water transportation and improve the evaporation efficiency,^[^ ^13^, ^15^ ^]^ making them promising options for WEIGs materials. Polyacrylonitrile (PAN) is widely considered an ideal electrospinning substrate due to its exceptional mechanical properties, spinnability, and stability.^[^ ^16^ ^]^ However, PAN nanofibers lack charge carriers and thus cannot be directly used for water‐evaporation power generation. This issue is typically addressed by carbonizing the fibers into carbon nanofibers, which introduces charge carriers, but this process results in the loss of flexibility.^[^ ^11^ ^]^ Therefore, in this work, we introduce lignin and zinc oxide into PAN nanofiber as solutions to this issue. Lignin, the second most abundant biopolymer on Earth, is rich in oxygen‐containing groups, which can enhance the polarity and hydrophilicity of the materials.^[^ ^17^ ^]^ Meanwhile, the in situ growth of ZnO nanoparticles on the nanofiber surface can increase the specific surface area and provide extra charge carriers.

**Figure 1:**
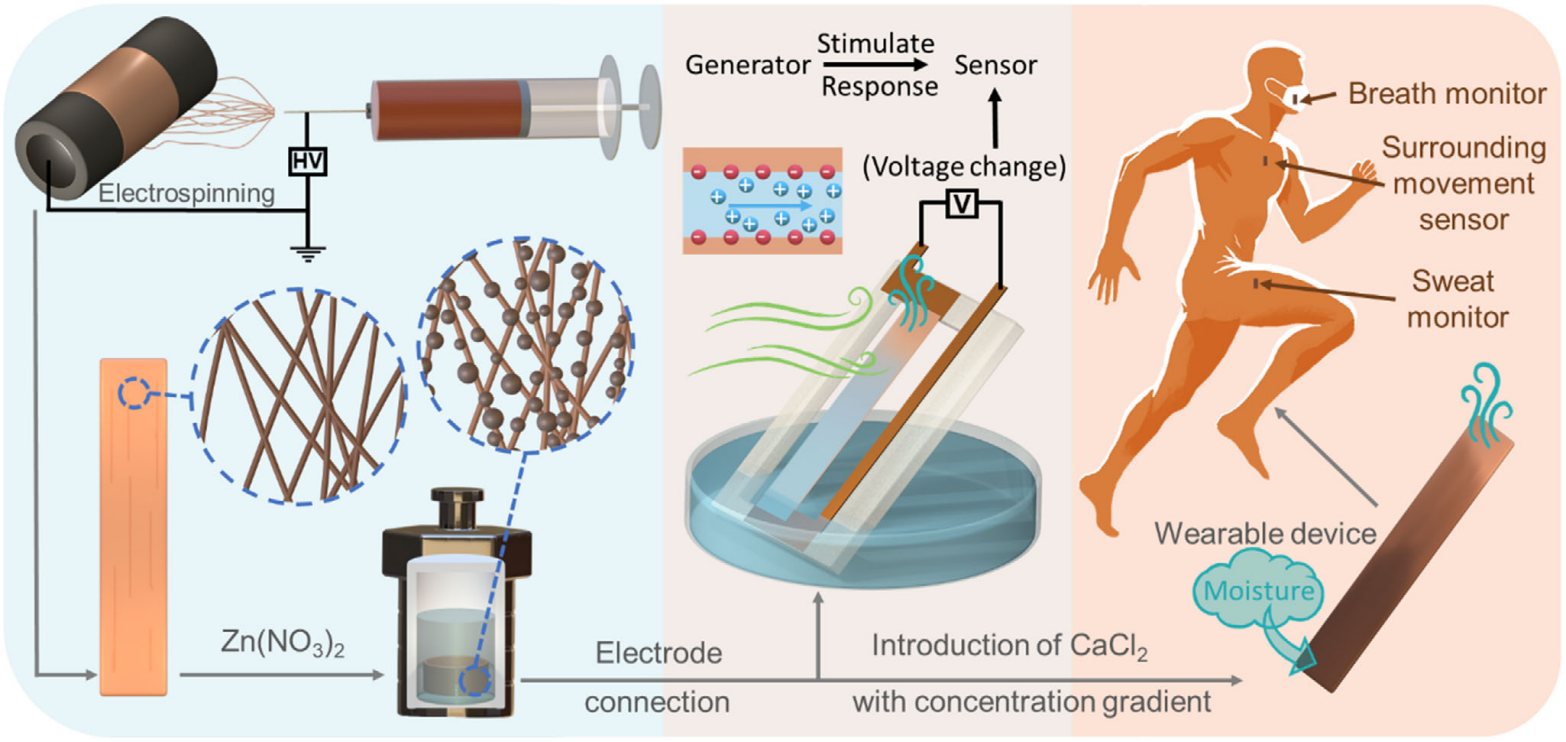
Schematic illustration of the fabrication process, working principle, and application of LP‐ZnO‐CaCl_2_‐NF membrane as a wearable device.

Herein, with further extension based on the mechanism of WEIGs, we designed a novel kind of self‐powered, ultrasensitive, flexible airflow sensor. As depicted in **Figure** **1**, the lignin‐PAN nanofiber (LP‐NF) was fabricated using electrospinning technology. Subsequently, ZnO nanoparticles were applied to the LP‐NF via a solvothermal reaction. The ZnO‐decorated LP‐NF (LP‐ZnO‐NF) was then connected to electrodes and partially immersed in water. The output voltage generated by the LP‐ZnO‐NF membrane will change in response to variations in water evaporation speed caused by modulating the flow of gas, enabling the LP‐ZnO‐NF membrane to function as an airflow sensor. Additionally, by incorporating calcium chloride (CaCl_2_) at one end of the membrane, the CaCl_2_ asymmetrically incorporated LP‐ZnO‐NF (LP‐ZnO‐CaCl_2_‐NF) membrane can absorb moisture from the air to maintain asymmetric wetting, thereby eliminating the dependence on liquid water sources. With such construction, the LP‐ZnO‐CaCl_2_‐NF membrane can be used to monitor the sweat, breath, and surrounding movement of the wearer.

## 2 Results and Discussion

**Figure 2:**
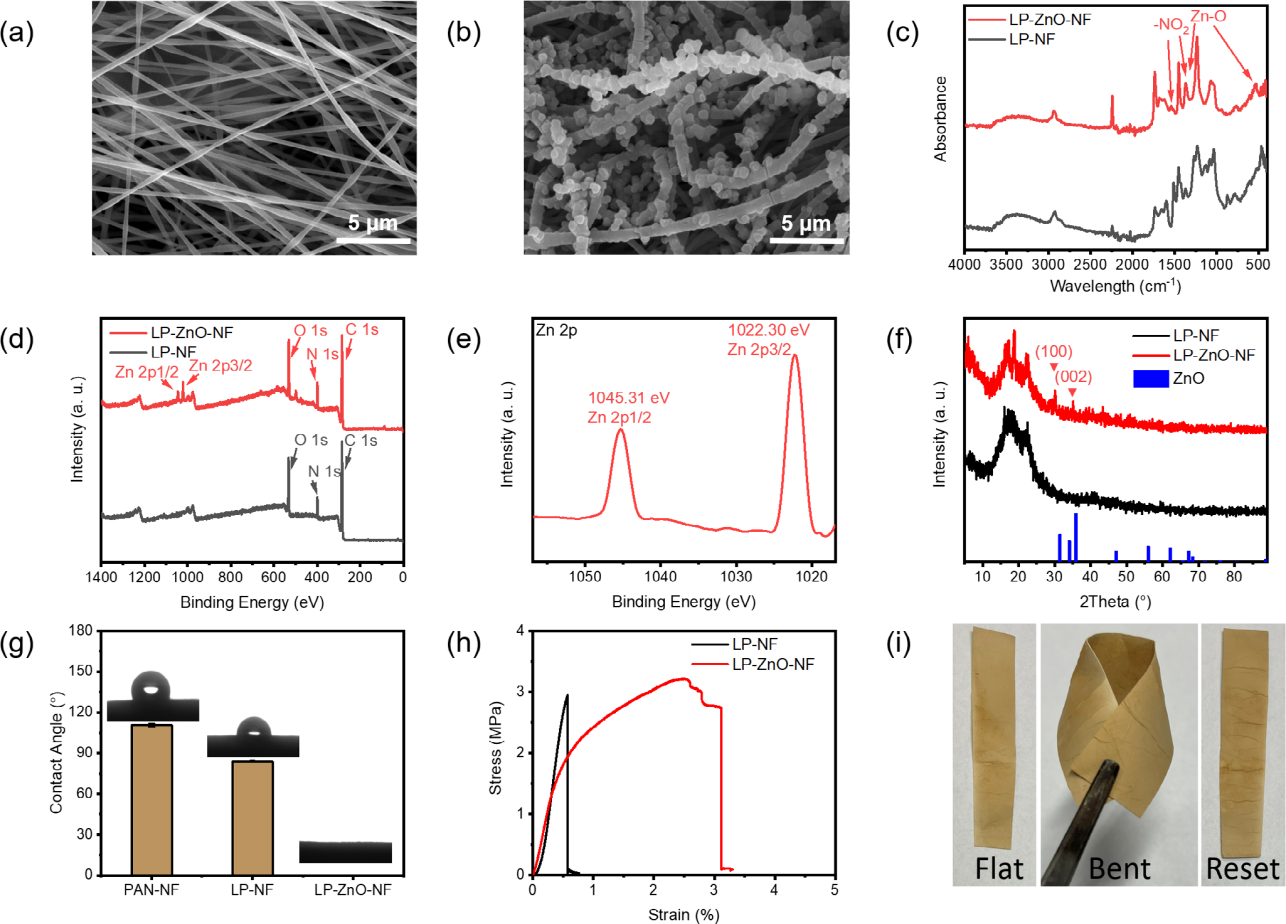
Structural, chemical and morphological characterization of prepared nanofibrous membranes. a) SEM image of LP‐NF. b) SEM image of LP‐ZnO‐NF. c) FTIR spectra of LP‐NF and LP‐ZnO‐NF. d) XPS spectra of LP‐NF and LP‐ZnO‐NF. e) Zn 2p XPS spectrum of LP‐ZnO‐NF. f) XRD diffractograms of LP‐NF and LP‐ZnO‐NF and PDF card of ZnO (JCPDS card 36‐1451). g) Static water contact angles of PAN‐NF, LP‐NF and LP‐ZnO‐NF (n = 3). h) Tensile stress‐strain curves of LP‐NF and LP‐ZnO‐NF (n = 3). i) Bending behavior of LP‐ZnO‐NF (n = 5).

As‐prepared nanofibers LP‐NF have an oriented fiber structure with an average diameter of 346 ± 73.5 nm in **Figures** **2**a and S1a (Supporting Information). The diameter of the nanofibers increases to 703 ± 99.2 nm upon introduction of the ZnO layer (Figure 2b; Figure S1b, Supporting Information). ZnO nanoparticles can be observed on the surface of the nanofibers after the growth of the ZnO (Figure 2b). Meanwhile, preferential orientation is observed in both LP‐NF and LP‐ZnO‐NF (Figure S1c, Supporting Information). Additionally, the elemental mapping images of the LP‐ZnO‐NF membranes in Figure S2 (Supporting Information) confirm the uniform distribution of ZnO on the surface of the nanofibers.

The FTIR spectrum of the LP‐NF membranes in Figure 2c contains characteristic peak of both the nitrile group in PAN at 2241 cm^−1^ and the stretching vibrations of the C═C bonds on the aromatic rings of lignin from 1400–1650 cm^−1^ which indicates that the electrospinning process does not affect the chemical structure of PAN and lignin.^[^ ^18^ ^]^ The appearance of new peaks in the FTIR spectrum of LP‐ZnO‐NF membrane can be attributed to the reaction between zinc nitrate and lignin or PAN during the solvothermal reaction including the incorporation of nitro groups in the products (1540 and 1369 cm^−1^) and the growth of ZnO on the surface of nanofibers (1369 and 537 cm^−1^).^[^ ^19^ ^]^ Meanwhile, the intensification of the peak at 2241 cm^−1^ is attributed to the partial dissolution of the lignin during the solvothermal reaction which increases the content of PAN. The presence of Zn is further validated via XPS spectrum (Figure 2d). The Zn 2p spectrum of LP‐ZnO‐NF in Figure 2e illustrates the Zn 2p3/2 and Zn 2p1/2 peaks of LP‐ZnO‐NF located at 1022.3 and 1045.3 eV, and the 23 eV energy difference is in good agreement with the reported value of Zn^+2^ oxidation state.^[^ ^20^ ^]^ Moreover, the C 1s and N 1s spectra of the LP‐NF and LP‐ZnO‐NF membranes in Figure S3 (Supporting Information) indicate that the lignin and PAN macromolecules do not undergo significant chemical changes before and after the solvothermal reaction.^[^ ^21^ ^]^ The XRD patterns of LP‐NF and LP‐ZnO‐NF membranes both show diffraction peaks at 2*θ* = 16.8° that are attributed to the crystalline structure of PAN (Figure 2f).^[^ ^22^ ^]^ The peaks of LP‐ZnO‐NF membrane at 30.1 and 35.1° are corresponding to the (1 0 0) and (0 0 2) planes of ZnO, respectively (JCPDS card 36–1451).^[^ ^23^ ^]^ Hence, ZnO is present in the shape of nanospheres in the LP‐ZnO‐NF membranes as verified with elemental mapping, FTIR, XPS and XRD.

To investigate the effect of adding lignin and ZnO, the static water contact angles (WCAs) of the LP‐NF membranes, LP‐ZnO‐NF and the pure PAN nanofibrous membranes (PAN‐NF) were measured. Although PAN contains polar groups, such as nitrile groups, their fine diameter and high surface roughness result in a static WCA of ≈110.5 ± 1.12° as shown in the Figure 2g.^[^ ^24^ ^]^ The incorporation of lignin enhanced the hydrophilicity of the PAN nanofibers, reducing their static WCA to 83.8 ± 0.39° due to the multiple oxygen‐containing functional groups in lignin. The ZnO modification endows the nanofibrous membrane with superhydrophilicity. The water droplets were completely absorbed by the membranes within 1 s which is highly beneficial for the transport of water through the nanofibrous membranes.

The toughness of the nanofibrous membranes increased from 4.91 ± 0.09 to 61.03 ± 19.69 MJ m^−3^ following ZnO growth modification, as illustrated in Figures 2h and S4 (Supporting Information), due to the possible cross‐linking during the solvothermal reaction. Furthermore, the absence of cracks after bending the nanofibrous membranes is indicative of its flexibility as shown in Figure 2i. Therefore, the enhanced toughness and flexibility of the membrane ensures its stability when used as a wearable device.

**Figure 3:**
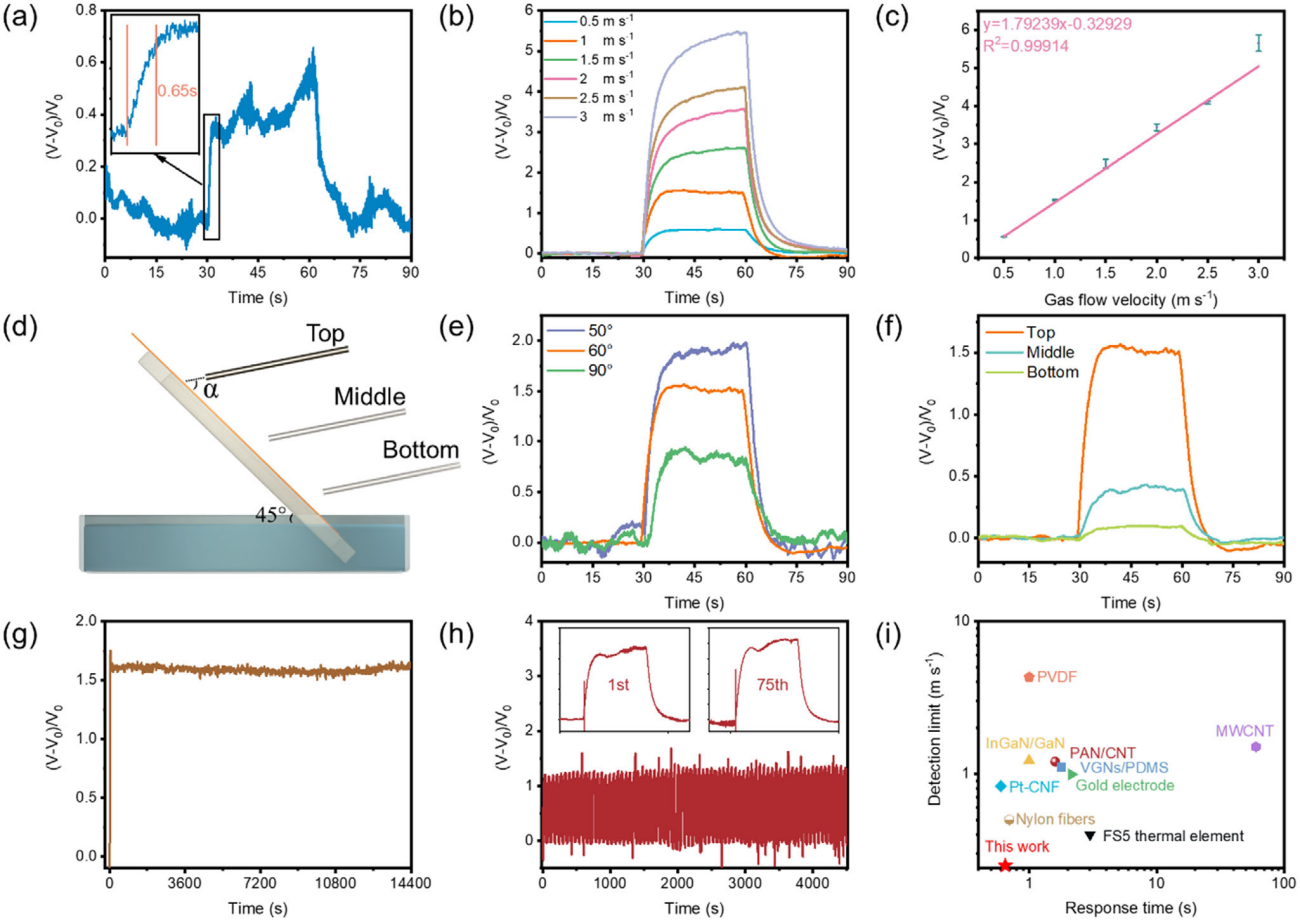
Airflow response performance of LP‐ZnO‐NF airflow sensor. a) Determination of the response time defined as the time taken to reach the voltage variation of 90% from 0% when gas flow (0.5 m s^−1^) is applied (n = 3). b) The response curve of LP‐ZnO‐NF airflow sensor with different gas flow velocities (The gas flow angle is 60° and the gas flow is blowing at the top region of the membrane) (n = 3). c) Linear fit of the relative voltage changes versus gas flow velocity (n = 3). d) Schematic diagram of the setup for the gas flow sensing test. e) The response curve of LP‐ZnO‐NF airflow sensor with different gas flow (1 m s^−1^) angles (n = 3). f) The response curve of LP‐ZnO‐NF airflow sensor with different gas flow (1 m s^−1^) blowing positions (n = 3). g) The long‐term sensing process of LP‐ZnO‐NF airflow sensor for over 4 h (1 m s^−1^) (n = 3). h) The response curve of LP‐ZnO‐NF airflow sensor with 75 gas flow (1 m s^−1^) on‐off cycles (n = 3). i) Comparison of response time and detection limit of LP‐ZnO‐NF airflow sensor and the reported airflow sensors in refs.[25]

In the absence of surrounding gas flow, the LP‐ZnO‐NF airflow sensor can generate a voltage of ≈105 mV, as shown in Figure S5 (Supporting Information). We refer to the voltage variation ((*V*‐*V_0_*)/*V_0_* (%)) as the response intensity and tested the response of the LP‐ZnO‐NF airflow sensor toward the nitrogen gas flow under different conditions to explore the performance of the LP‐ZnO‐NF as airflow sensor, where *V_0_* and *V* represent the output voltage without gas flow and the real‐time output voltage, respectively. To accurately determine the response time and avoid the influence of gas acceleration, a plastic plate was placed between the LP‐ZnO‐NF airflow sensor and the outlet of a nitrogen gas source with a continuous flow, and is removed during testing. **Figures** **3**a and S6a (Supporting Information) show that the time required for the LP‐ZnO‐NF airflow sensor to reach 90% of the voltage variation is about only 0.65 s (referred to as the response time). Figures 3b and S6b (Supporting Information) show the response behavior of the LP‐ZnO‐NF airflow sensor at various gas flow rates from 0.5 to 3 m s^−1^, and the LP‐ZnO‐NF airflow sensor shows a stable signal after reaching stable gas flow. A clearly strong linear correlation between the voltage variation and gas flow velocity within the testing range was recorded, as shown in Figure 3c. The sensitivity of the LP‐ZnO‐NF airflow sensor, which is determined from the slope of the voltage variation and the gas flow velocity, is 1.79% (m s^−1^)^−1^. It is worth noting that the detection threshold can be lowered to 0.25 m s^−1^ by adjusting the outlet area of the gas flow tube. Based on favorable and linear response to gas flow velocity, the optimal operating range for the LP‐ZnO‐NF airflow sensor is ≈0.25‐3 m·s^−1^ (Figure 3; Figure S7, Supporting Information).

The sensing properties of PAN‐NF, ZnO decorated PAN nanofibrous membranes (PAN‐ZnO‐NF) and LP‐NF were compared to assess the effects of incorporated lignin and ZnO. As shown in Figure S8 (Supporting Information), the pure PAN membrane demonstrates no response to gas flow, as it cannot generate power through water evaporation. Both the PAN‐ZnO‐NF and LP‐NF membranes respond to 1 m s^−1^ gas flow, but their responses are rather slow that even cannot complete within one minute. This observation demonstrates that the addition of lignin and ZnO enhances water transport and provides sufficient charge carriers for power generation. To investigate the critical role of water evaporation in the airflow sensing behavior of the LP‐ZnO‐NF airflow sensor, we sealed the sensor with plastic wrap to prevent evaporation. As shown in Figure S8d (Supporting Information), once encapsulated, the LP‐ZnO‐NF airflow sensor exhibited no response to airflow stimulation, clearly demonstrating that its airflow sensing capability is driven by water evaporation. Additionally, the LP‐ZnO‐NF airflow sensor shows minimal sensitivity to gas composition – including N_2_, O_2_, and air – which indicates that it is more suitable for general airflow sensing rather than gas‐specific detection (Figure S9, Supporting Information).

The sensing performance regarding the gas flow angles and different nitrogen source outlet positions of the LP‐ZnO‐NF airflow sensor is further analyzed. As illustrated in the setup (Figure 3d), α denotes the angle between the nitrogen gas flow and the LP‐ZnO‐NF membrane. The LP‐ZnO‐NF airflow sensor is sensitive to the incident angle of gas flow. The lower the α is, the higher is the response generated by the LP‐ZnO‐NF airflow sensor (Figure 3e; Figure S6c, Supporting Information). This is due to the inherent dependence on the water evaporation in the membrane promoted by gas flow. The membrane absorbs energy in the vertical direction of the gas flow, resulting in a reduction in gas flow velocity. In addition to the gas flow angle, the LP‐ZnO‐NF airflow sensor shows a spatial dependence (Figure 3f; Figure S6d, Supporting Information). The top region of the LP‐ZnO‐NF airflow sensor shows a higher response intensity compared to the middle and the bottom region of the LP‐ZnO‐NF airflow sensor, which also indicates a higher sensitivity at this top position. This is because the top region of the sensor is farther from the water source, making it more difficult for the water flow to replenish the evaporated water. Consequently, when exposed to gas flow of the same velocity, the moisture content in the top region is more easily altered, thereby causing greater changes in the generated voltage.

The effect of the structure of the LP‐ZnO‐NF membrane was further investigated. We tested the response behavior of LP‐ZnO‐NF membranes with varying fiber diameters to a 1 m s^−1^ gas flow. The different fiber diameters had minimal impact on the response behavior (Figure S10a, Supporting Information), likely because water evaporation primarily occurs on the surface of the membrane, and the change in fiber diameter does not significantly affect the surface area of the surface layer. As comparison, when using a flat plate collector instead of a drum collector for while electrospinning, the resulting nanofibers were randomly distributed rather than oriented along an axis. Membranes with randomly distributed nanofibers respond more slowly and weakly (Figure S10b, Supporting Information). Hence, an oriented distribution of nanofibers within the airflow sensor facilitates the water transport and thereby enhances the responsiveness.

Furthermore, a constant gas flow was applied to test the response stability of the LP‐ZnO‐NF airflow sensor. The sensor shows a stable and continuous response for 4 h without any fluctuation, indicating the stability of the sensor as shown in Figure 3g. Additionally, cyclic reliability measurement was also carried out by periodically turning on/off the gas flow every 30 s. As shown in Figure 3h, the response of the LP‐ZnO‐NF airflow sensor remains stable during 75 consecutive cycles without any obvious degradation, and the 75th response shows a similar response to that of the first cycle, further confirming the stability and reliability of the LP‐ZnO‐NF airflow sensor.

Figure 3i compares the response time and the detection limit of the prepared LP‐ZnO‐NF airflow sensor with those of previously reported airflow sensors from the last five years.^[^ ^25^ ^]^ These reported airflow sensors based on versatile sensing principles include piezoelectric, piezoresistive, piezo‐resistive mechanoreceptors, thermal convection, hot wire and hot film as listed in Table S1 (Supporting Information). Different than the other airflow sensors, using a WEIG as an airflow sensor is based on voltage change induced by water‐evaporation. As‐prepared LP‐ZnO‐NF airflow sensor not only achieves a rapid response (0.65 s) and a low detection limit (0.25 m s^−1^), but also can self‐power by absorbing water flow from a water source. Consequently, the sensor we developed surpasses previously reported airflow sensors in performance and demonstrates excellent potential for further application.

**Figure 4:**
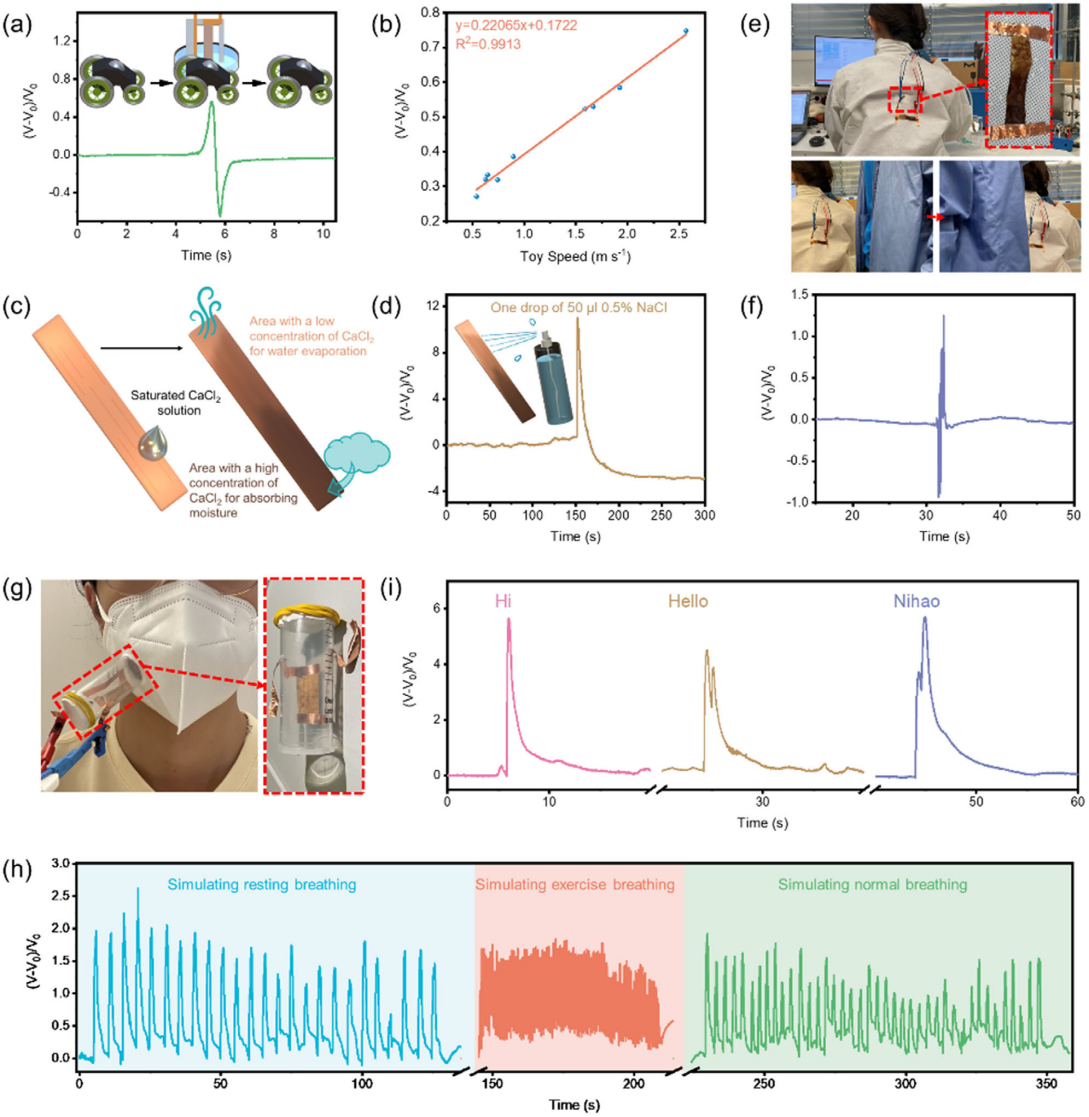
Application of the lignin/ZnO airflow sensor. a) Schematic diagram and response curve of LP‐ZnO‐NF airflow sensor to a toy car passing by. b) Linear fit of the relative voltage changes versus toy car velocity. c) Schematic diagram of the preparation of the LP‐ZnO‐CaCl_2_‐NF membrane with CaCl_2_ concentration gradient. d) The response curve of LP‐ZnO‐CaCl_2_‐NF membrane to a spray of 0.5% NaCl solution simulating human perspiration. e) Photographs of LP‐ZnO‐CaCl_2_‐NF membrane as wearable device to sense the variation in airflow caused by people passing by. f) The response curve of the LP‐ZnO‐CaCl_2_‐NF membrane as wearable devices to a person passing by. g) Photographs of the LP‐ZnO‐CaCl_2_‐NF membrane in wearable devices for breath monitoring. h) The response curve of the LP‐ZnO‐CaCl_2_‐NF equipped mask towards three different simulated breathing conditions (resting, exercising, and normal activity). i) The response curve of LP‐ZnO‐CaCl_2_‐NF equipped mask to the breath of pronouncing three different words, “Hi”, “Hello”, and “Nihao”.

The LP‐ZnO‐NF airflow sensor can be used to detect the movement around the sensor due to its ability to sense variation in airflow. Herein, we used a remote‐controlled toy car driving past the LP‐ZnO‐NF airflow sensor. The sensor can sense the passing of the toy car and the response can be observed as shown in **Figure** **4**a and Movie S1 (Supporting Information). The response results from the change airflow induced by the toy car. Moreover, the magnitude of the response peak is linearly related to the speed of the toy car, as illustrated in Figure 4b. This relationship endows the LP‐ZnO‐NF airflow sensor with the potential to function as a self‐powered speedometer in some specific areas, such as maritime and environmental monitoring.

Furthermore, the incorporation of the certain hydroscopic compounds such as CaCl_2_, into the membrane promotes ambient moisture adsorption on the deposition side and the evaporation of the moisture on the other. Figure 4c illustrates the addition of CaCl_2_ and the process of its moisture adsorption and evaporation. The CaCl_2_ can be easily incorporated into the LP‐ZnO‐NF membrane with a certain gradient after drop‐casting the aqueous CaCl_2_ solution onto one end of the membrane and drying it under vacuum. The incorporation of CaCl_2_ imparts the membrane with strong moisture absorption capability. Dynamic vapor sorption (DVS) results (Figure S11a, Supporting Information) reveal that the maximum water uptake increases significantly from ≈10% to nearly 400% after the introduction of CaCl_2_. This substantial enhancement effectively eliminates the dependence of the LP‐ZnO‐NF airflow sensor on liquid water sources, thereby expanding its potential for application as a wearable sensor device. The gradient of CaCl_2_ content in the membrane was validated by cutting the membrane into fragments at different positions, immersing them in DI water, and testing the conductivity of the resulting solutions. The variation in conductivity verified the existence of the CaCl_2_ content gradient (Figure S12, Supporting Information). In addition, the LP‐ZnO‐CaCl_2_‐NF membrane exhibits a more intense response to gas flow under higher humidity conditions (Figure S11b, Supporting Information), which can be attributed to the greater humidity gradient generated when dry nitrogen flows over a sample pre‐conditioned in a high‐humidity environment. This suggests that the LP‐ZnO‐CaCl_2_‐NF membrane holds greater potential for applications in high‐humidity environments.

Given that the power generation mechanism of the WEIG is based on charge transfer, changes in charge concentration within the generator system will directly affect the voltages produced. This endows the wearable device with the potential to monitor human perspiration. As shown in Figure 4d, the sweat monitor test was performed by spraying 0.5% NaCl solution on the LP‐ZnO‐CaCl_2_‐NF membrane to simulate human sweat, and the response curve showed a strong peak after the spray due to the airflow variation caused by the spray bottle, then the baseline of the response curve stabilizes at a lower level after the airflow is removed and the solution is absorbed by the membrane. Using this airflow sensor on clothes (Figure 4e), the LP‐ZnO‐CaCl_2_‐NF membrane can be used as wearable device to detect the movement. A sharp response peak is observed when people walk past the sensor and quickly recovers upon removal of the stimulus (Figure 4e,f). This function of the LP‐ZnO‐CaCl_2_‐NF membrane enables it to alert wearers with visual impairments or those in low‐light or obstructed‐view environments to movements in their surroundings. This capability could help to avoid potential collisions and ensure the personal safety of wearers.

Based on the mechanical flexibility, the LP‐ZnO‐CaCl_2_‐NF membranes can also be incorporated into a mask to monitor the breathing behavior of the wearer. As depicted in Figure 4g, the LP‐ZnO‐CaCl_2_‐NF membrane was adhered on the inner surface of a plastic tube sealed by the filtered paper, and the plastic tube was then attached to a mask. Figure 4h illustrates the response curve of the LP‐ZnO‐CaCl_2_‐NF equipped masks with three different breathing behaviors of the wearer, which are resting breathing (12 to 15 breaths per minute), exercise breathing (more than 60 breaths per minute) and normal breathing (≈20 breaths per minute). The LP‐ZnO‐CaCl_2_‐NF equipped mask accurately monitors each breath and depicts them with distinct response peaks, whether the breath lasts 5 seconds or less than 0.8 seconds. Moreover, since the pronunciation of different words leads to variations in respiratory patterns, the sensor can roughly monitor the wearer's pronunciation behavior through their breathing behaviors. In order to test the pronunciation monitoring ability, the wearer pronounced three greeting words with different phonetic dynamics which were Hi, Hello and Nihao (a greeting in Chinese). The response curve of the LP‐ZnO‐CaCl_2_‐NF equipped mask shows three different but related shapes (Figure 4i), although the LP‐ZnO‐CaCl_2_‐NF equipped mask can only detect the airflow generated when speaking. While the device lacks the precision of commercial speech recognition tools, it already shows promising potential to assist in recognition tasks.

## 3 Conclusion

In summary, inspired by the mechanism of water‐evaporation‐induced power generation, we designed a self‐powered, ultrasensitive flexible airflow sensor. The prepared airflow sensor exhibited an ultrafast response time (0.65 s), broad detection range (with lower detection limit down to 0.25 and upper detection limit of 3 m s^−1^) and accuracy toward airflow speed testing. Additionally, the sensor demonstrated outstanding long‐term monitoring stability (4 h) and multiple‐cycle detection reliability (75 cycles). Furthermore, we optimized the LP‐ZnO‐NF membrane to function as a multifunctional self‐powered wearable sensor, capable of monitoring the wearer's perspiration, surrounding movement, and respiratory conditions. The prepared sensor exhibited superior performance, good flexibility, and ease of fabrication, which demonstrate a promising prospect in wearable electronics and beyond.

## 4 Experimental Section

### Materials

Polyacrylonitrile (PAN, *Mw* 150 000 g mol^−1^) was purchased from BLD Pharmatech GmbH, Kraft lignin (*Mw* 5000 g mol^−1^) was purchased from UPM Biochemicals, zinc nitrate hexahydrate, and calcium chloride were purchased from Thermo Fischer Scientific, sodium chloride, 1,4‐dioxane and N,N‐dimethylformamide (DMF) was purchased from ChemSolute.

### Fabrication of the LP‐NF

PAN and lignin (2:3 w:w) were stirred in DMF at 60 °C for 4 h to get a 15% homogeneous solution. After cooling, 10 mL of the mixed solution was transferred into a syringe equipped with a spinning needle with an inner diameter of 0.8 mm. Electro‐spinning was then performed with a voltage of 20.0 kV and a distance of 15 cm between the spinning needle and the rolling collector with a diameter of 13 cm and the rolling speed of 500 rpm. The solution pumping rate was 1.0 mL h^−1^ and the total fiber collection time was 10 h. The nanofibrous membrane was then dried in a vacuum oven at 60 °C overnight. The dried nanofibrous membrane was then cut into smaller pieces with a size of 2.5 × 7.5 cm^2^, denoted as LP‐NF.

### Fabrication of LP‐ZnO‐NF and Airflow Sensor

The LP‐NF was immersed into a solution of zinc nitrate in 1,4‐dioxane (100 mg mL^−1^) in a Teflon‐lined reactor and solvothermally reacted at 120 °C for 2 h for the in situ growth of ZnO. The resulting membrane was then washed with deionized water (DI water) several times, dried at 60 °C overnight, and cut into pieces with a size of 1.5 × 6.5 cm^2^, denoted as LP‐ZnO‐NF. The prepared nanofibrous membrane was then adhered on a square silicone sheet with a hole in the center by copper tape and partly immersed in the water for the airflow sensing test, denoted as LP‐ZnO‐NF airflow sensor. Furthermore, 0.5 mL of 2.5 M CaCl_2_ solution was added dropwise to one end of the LP‐ZnO‐NF membrane (1 × 4 cm^2^), which was then placed vertically in an oven at 60 °C for drying, denoted as LP‐ZnO‐CaCl_2_‐NF membrane.

The experiments involving human subjects were carried out with the full, informed consent of the volunteers, who were also co‐authors of the paper. This study does not involve any ethical issue.

### Characterization

The surface morphology of the nanofibrous membrane was characterized by scanning electron microscopy (SEM) using a Nova NanoSEM 650 (American FEI Co.) at an accelerating voltage of 15 kV. The elemental mapping was checked by an Oxford Xmax 80 EDS system with INCA software. A layer of gold was coated on the surface of samples before SEM measurements. Fourier transform infrared spectroscopy (FTIR) was conducted by Bruker Alpha FTIR Spectrometer (Bruker, Germany) at room temperature. All nanofibrous membrane samples were measured between 4000 and 400 cm^−1^ with a resolution of 4 cm^−1^ by using Platinum ATR and accumulated 24 scans. The chemical surface compositions were analyzed with X‐ray photoelectron spectroscopy (XPS) using a PHI VersaProbe II (Ulvac‐phi, Inc., Chigasaki, Japan) with a monochromatic Al‐Kα source. X‐ray scattering patterns were recorded at ambient temperature using a Bruker D8 Discovery (X‐ray wavelength  =  1.54 Å, Cu Kα radiation). The static water contact angle was assessed by KRÜSS ADVANCE (KRÜSS) with the sessile droplet method. A 2.0 µl droplet was dropped on the nanofibrous membrane and the results were recorded every 1 s and analyzed through the ADVANCE software. Tensile test was tested by cutting the samples into a 30 × 10 mm^2^ plate and stretching the sample plate at a speed of 1 mm min^−1^ with a clamp distance of 15.0 mm by a tensile testing machines Z3 (Thümler GmbH) with a 50 N sensor. The open circuit voltage (OCV) was tested by potentiostat SP‐300 (Bio‐Logic SAS) with the OCV mode. The *dt_R_* was 0.01 s and the results were collected by calculating the average of every two adjacent points. A DVS apparatus (DVS Advantage, Surface Measurement Systems) was used to assess the dynamic water vapor sorption behavior of all samples.

### Statistical Analysis

All data were displayed as the mean ± standard deviation (*n* ≥ 3). Data analyses were performed using Origin (2020). The significant difference between the groups was determined via t‐test with the critical α‐level set at p < 0.05. and the significant levels were denoted as *p < 0.05, **p < 0.01, and ***p < 0.001.

## Conflict of Interest

The authors declare no conflict of interest.

## Supporting information



Supporting Information

Supplemental Movie 1

## Data Availability

The data that support the findings of this study are available from the corresponding author upon reasonable request.
