# Supplementary material for: Electrospun Lignin/ZnO Nanofibrous Membranes for Self‐Powered Ultrasensitive Flexible Airflow Sensor and Wearable Device
Source: Adv Mater. 2025 Jul 2;37(37):2502211. doi: 10.1002/adma.202502211 (PMC12447064; doi:10.1002/adma.202502211)
Supplement: Supplementary file 1 — Supporting Information [file ADMA-37-2502211-s002.docx]

Supporting Information

Electrospun Lignin/ZnO Nanofibrous Membranes for Self-powered Ultrasensitive Flexible Airflow Sensor and Wearable Device

Yifei Zhan, Jade Poisson, Xintong Meng, Zengbin Wang, Lizhen Chen, Tun-hui Wu, Robert Koehler, Kai Zhang*

Contents:

Supplementary Figures S1-S12.

Supplementary Table S1: Key parameters of the reported airflow sensors.

Supplementary Movie 1: Response behavior of LP-ZnO-NF airflow sensor to a toy car passing by.

Supplementary Figures


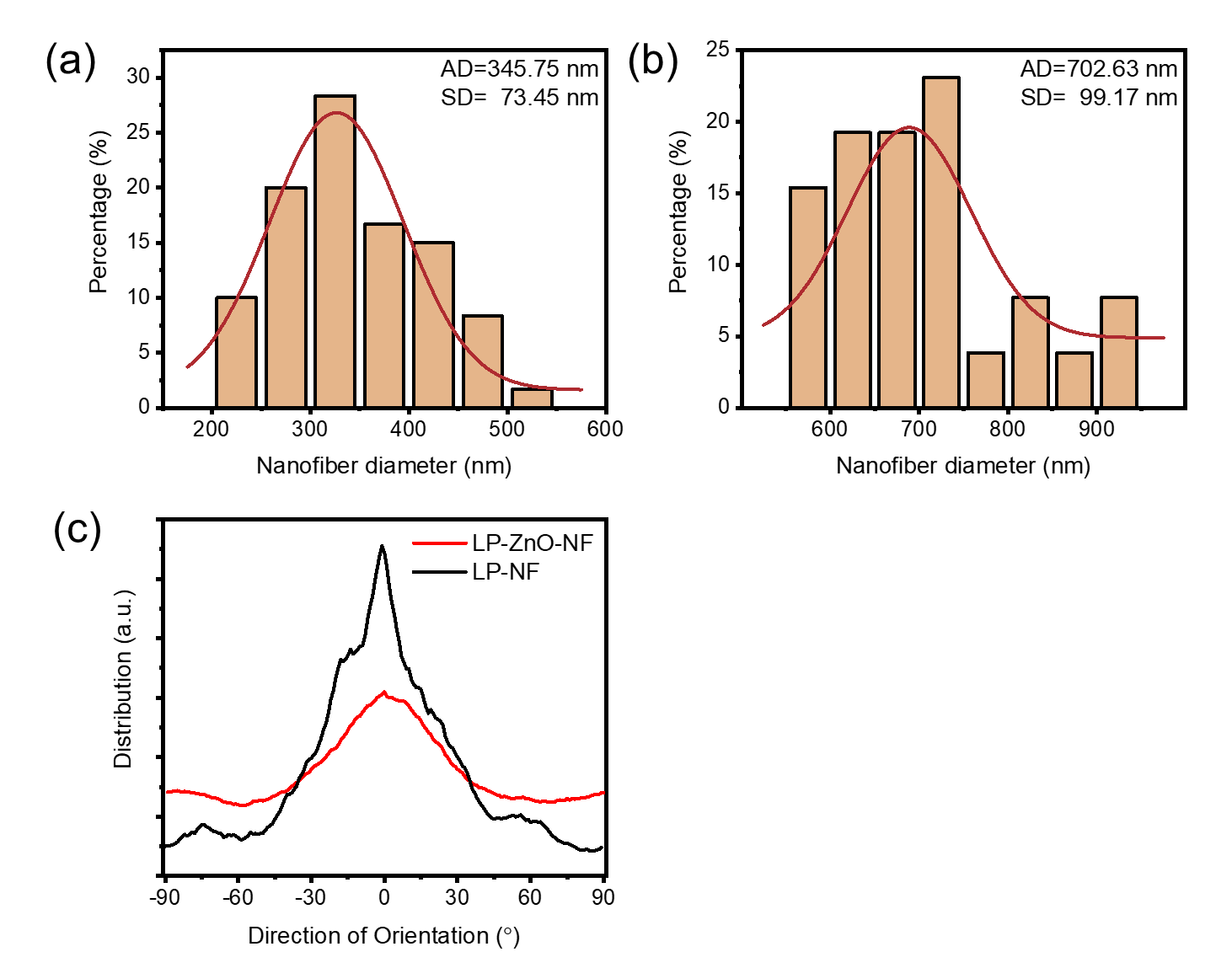


**Figure S1.** Diameter distribution of a) LP-NF and b) LP-ZnO-NF. c) Representative distribution of orientation of LP-NF and LP-ZnO-NF (n=3).


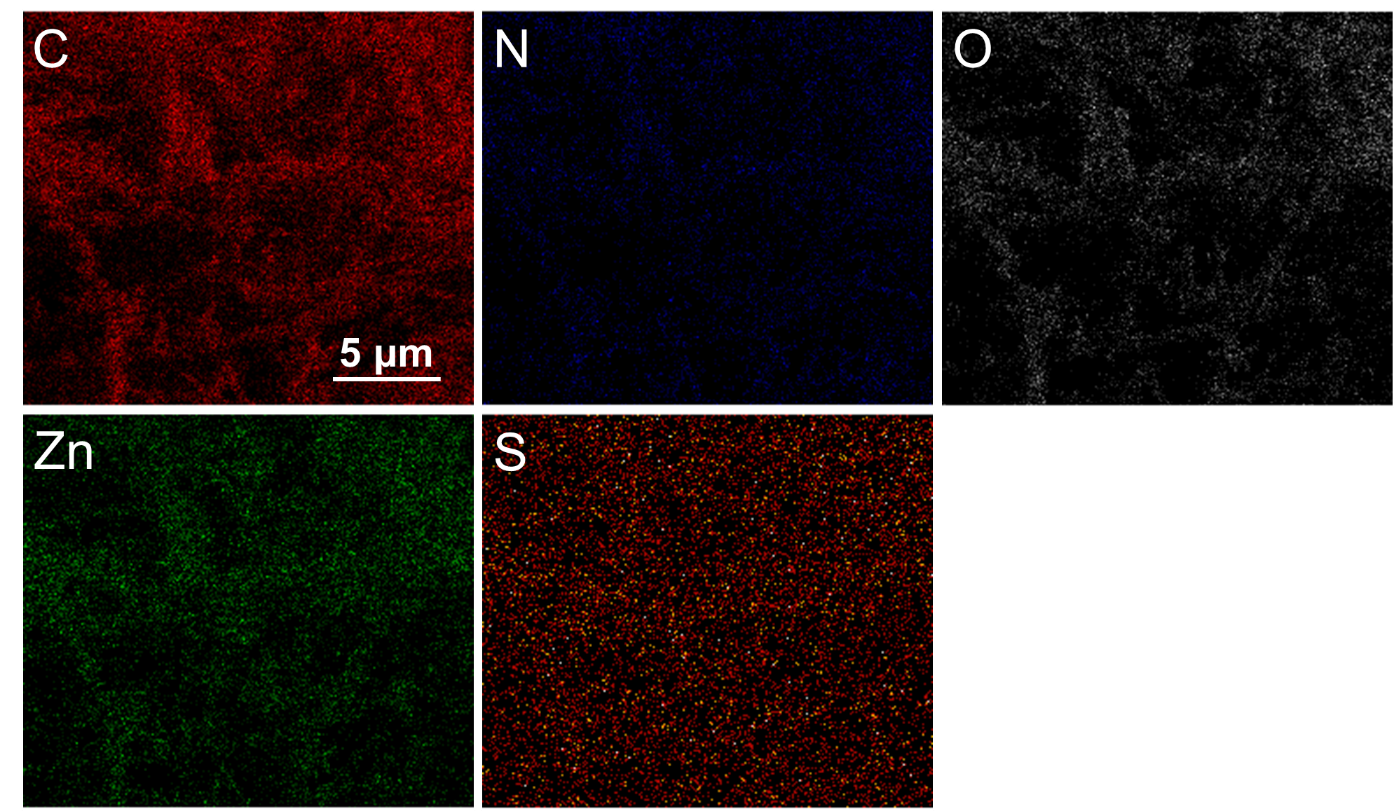


**Figure S2.** Elemental mapping images of LP-ZnO-NF.


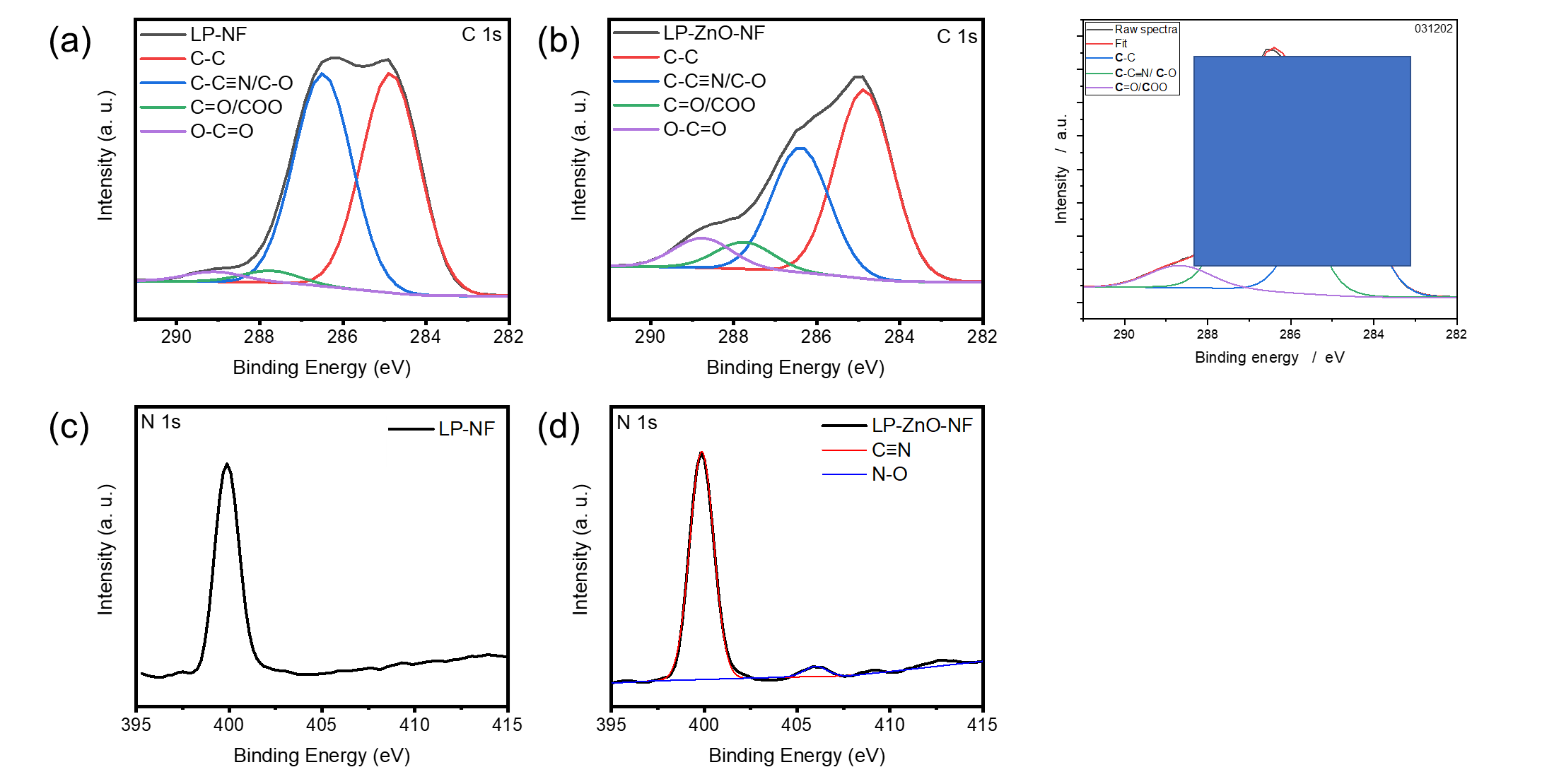


**Figure S3.** C 1s XPS spectra of a) LP-NF and b) LP-ZnO-NF. N 1s XPS spectra of c) LP-NF and d) LP-ZnO-NF.

**Figure S4.** The tensile strength and elongation at break of LP-NF and LP-ZnO-NF (n=3).


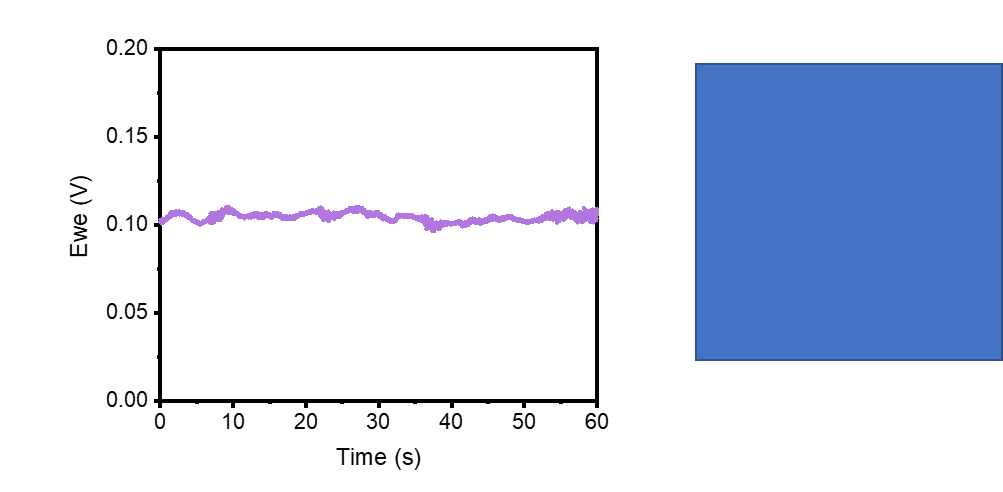


**Figure S5.** The generated voltage of LP-ZnO-NF airflow sensor in the absence of gas flow (n=3).


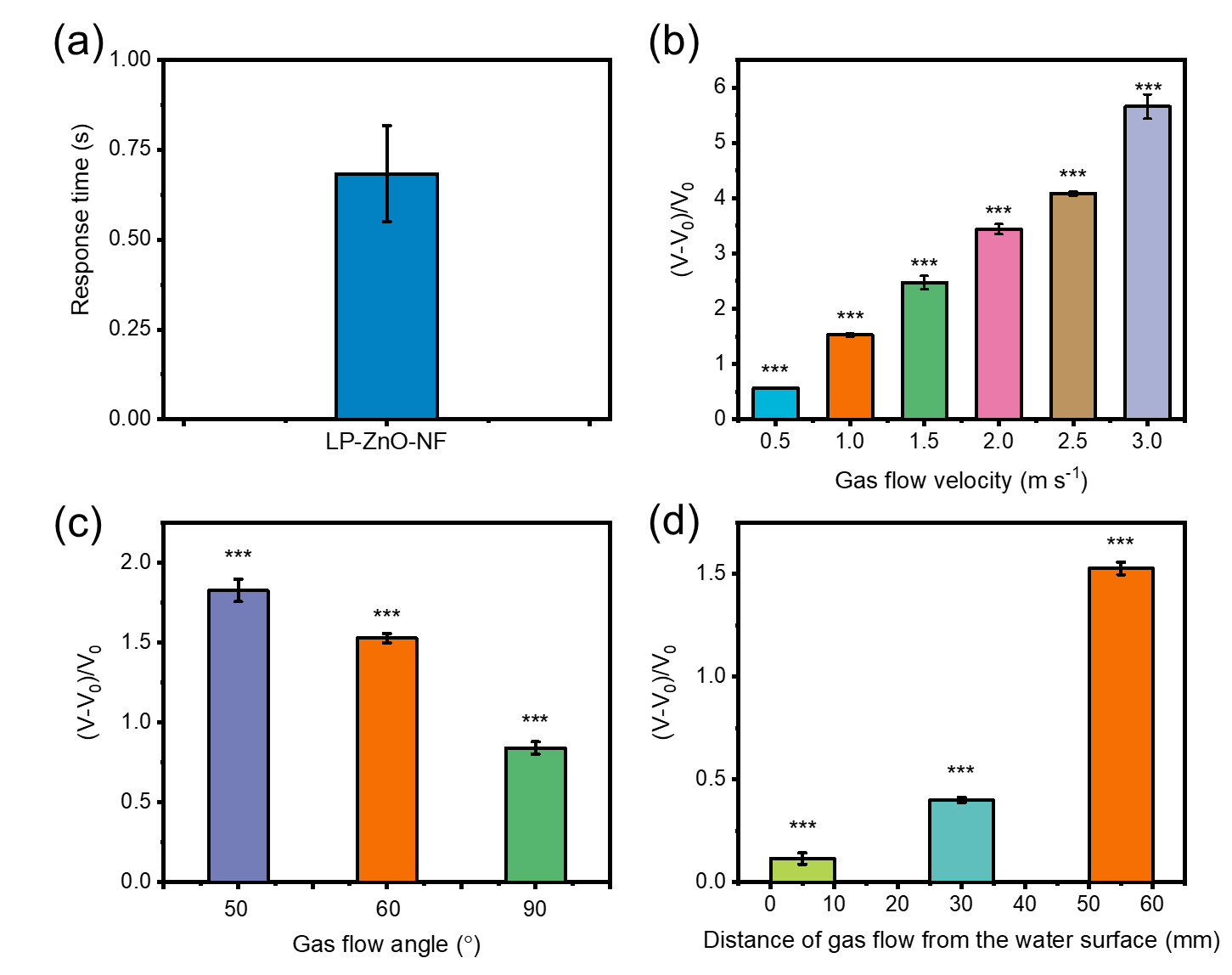


**Figure S6.** a) Response time of LP-ZnO-NF airflow sensor (n=3) towards (0.5 m s^-1^) gas flow. b) Response intensity of LP-ZnO-NF airflow sensor towards different gas flow velocity (n=3). c) Response intensity of LP-ZnO-NF airflow sensor with different gas flow (1 m s^-1^) angles (n=3). d) Response intensity of LP-ZnO-NF airflow sensor with different gas flow (1 m s^-1^) blowing positions (n=3). *** represents significant difference (p < 0.001).


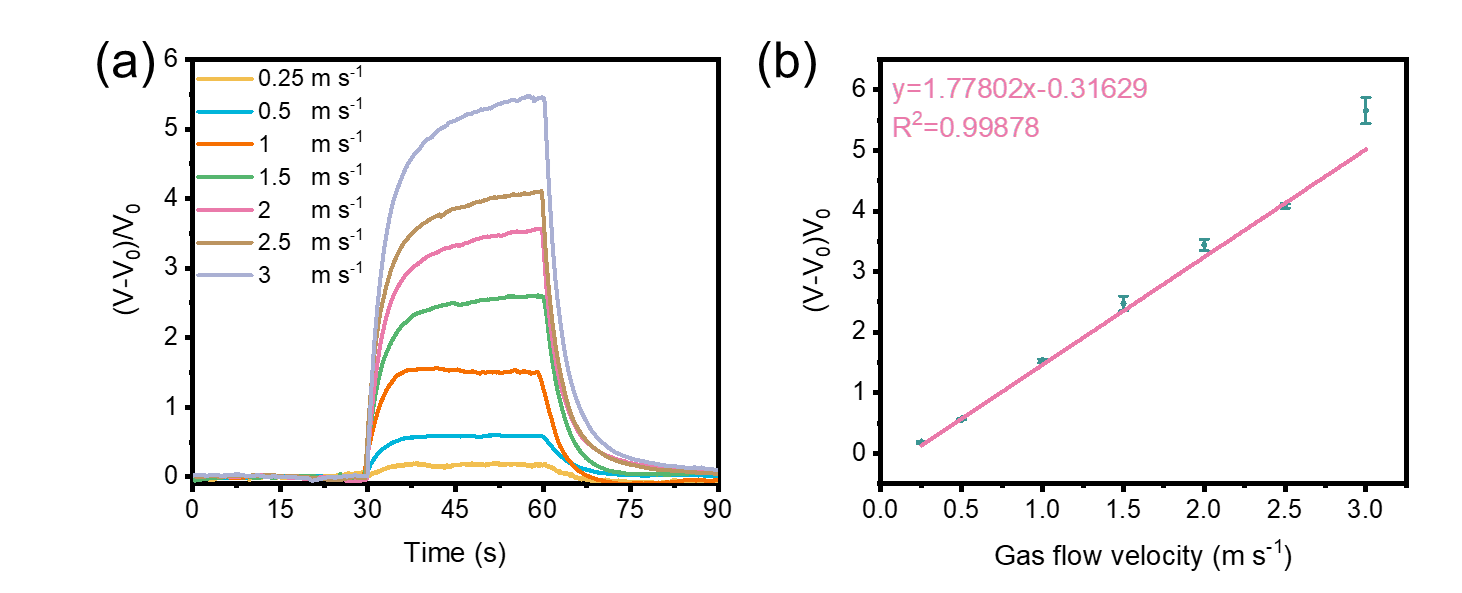


**Figure S7.** a) The response curve of LP-ZnO-NF airflow sensor with different gas flow velocities (0.25-3 m s^-1^) (the velocity of 0.25 m s^-1^ is obtained by adjusting the outlet area of the gas flow tube) (n=3). c) Linear fit of the relative voltage changes vs. gas flow velocity (n=3).


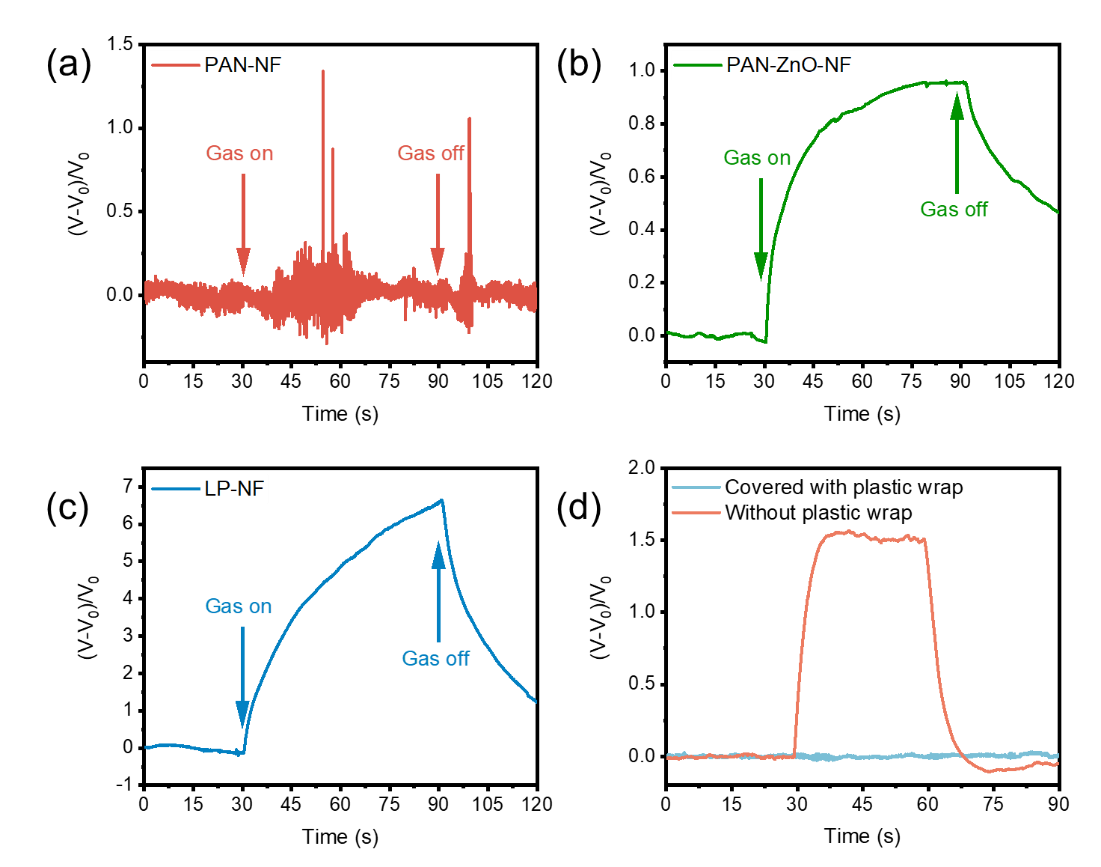


**Figure S8.** The response curves of a) PAN-NF b) PAN-ZnO-NF and c) LP-NF membrane to 1 m s^-1^ gas flow (n=3). d) The response curve of the LP-ZnO-NF airflow sensor with and without plastic wrap covered (n=3).

**Figure S9.** The response curve of LP-ZnO-NF airflow sensor towards different types of gases (1 m s^-1^) (n=3).


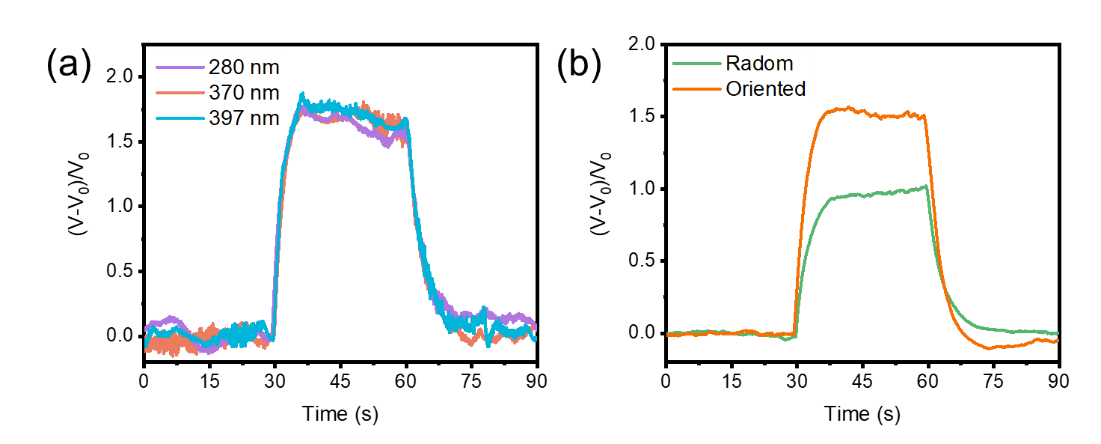


**Figure S10.** The response curves of LP-ZnO-NF membrane to 1 m s^-1^ gas flow. a) LP-ZnO-NF membrane with different nanofiber diameter. b) LP-ZnO-NF membrane with oriented nanofibers and randomly distributed nanofibers (n=3).


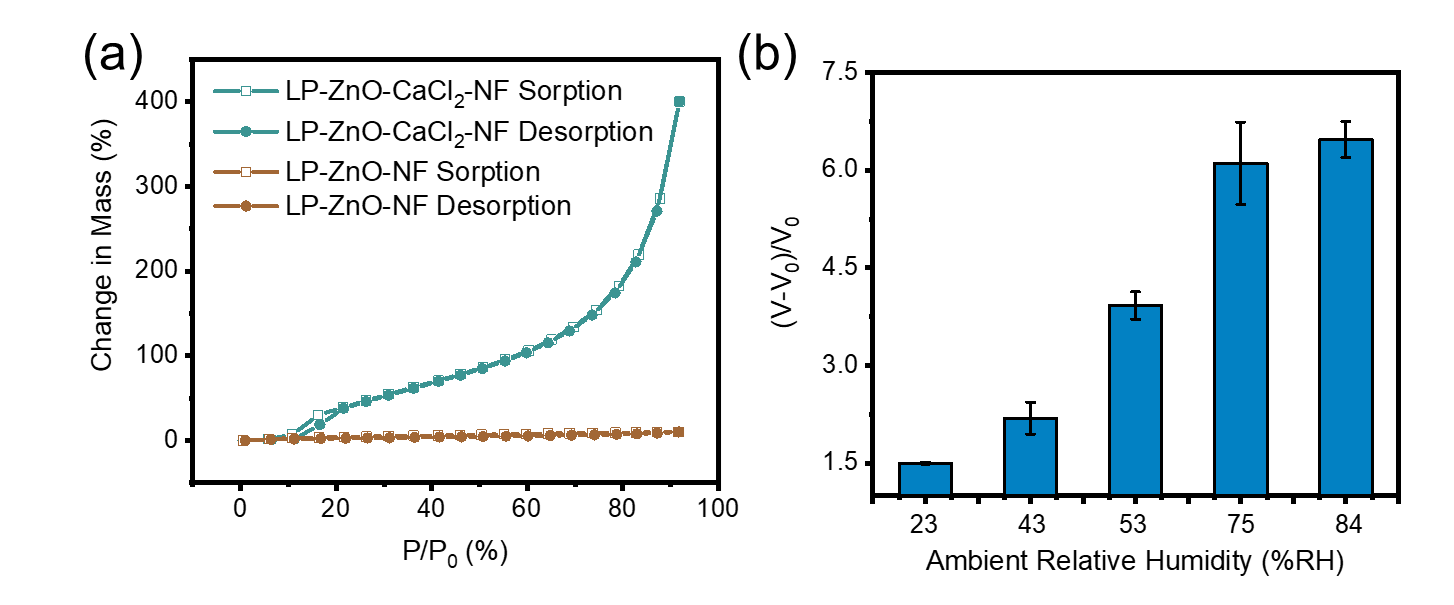


**Figure S11.** a) Water sorption and desorption isotherm of LP-ZnO-NF and LP-ZnO-CaCl_2_-NF membranes. b) Response intensity of LP-ZnO-CaCl_2_-NF membrane towards gas flow (1 m s^-1^) at different relative humidity (n=3).


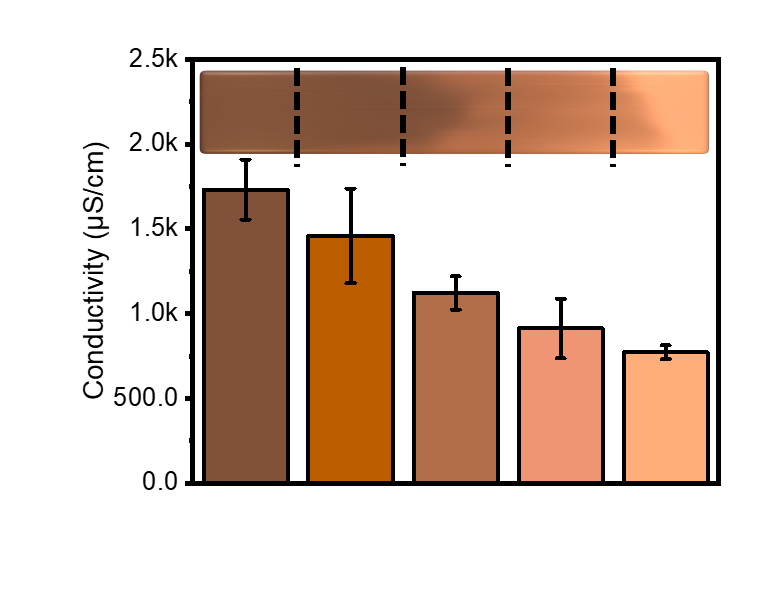


**Figure S12.** Conductivity of solutions from fragments of LP-ZnO-CaCl_2_-NF membrane with varying CaCl_2_ content (n=3).

Supplementary Table

**Table S1.** Key parameters of the reported airflow sensors.

| Ref | Materials | Principle | Response time [s] | Detection limit [m/s] |
| --- | --- | --- | --- | --- |
| [1] | Nylon fibers | Piezo-resistive mechanoreceptors | 0.7 | 0.5 |
| [2] | PAN/CNT | Hot wire | 1.6 | 1.2 |
| [3] | PVDF | Piezoelectric | 1 | 4.3 |
| [4] | VGNs/PDMS | Piezoresistance | 1.8 | 1.1 |
| [5] | Pt-CNF | Piezoresistance | 0.6 | 0.83 |
| [6] | InGaN/GaN | Piezoelectric | 1 | 1.222 |
| [7] | FS5 thermal element | Hot film | 3 | 0.4 |
| [8] | MWCNT | Piezoresistance | 60 | 1.5 |
| [9] | Gold electrode | Thermal convection | 2.15 | 0.99 |
| This work | Lignin | Water evaporation induced power generation | 0.65 | 0.25 |

**Reference**

[1] Y.-F. Liu, P. Huang, Y.-Q. Li, et al, A Biomimetic Multifunctional Electronic Hair Sensor, *Journal of Materials Chemistry A* **2019**, 7, 1889.

[2] T. Nguyen, T. Dinh, V. T. Dau, et al, A Wearable, Bending-Insensitive Respiration Sensor Using Highly Oriented Carbon Nanotube Film, *IEEE Sensors Journal* **2021**, 21, 7308

[3] J. Hu, H. Peng, T. Mao, et al, An Airflow Sensor Array based on Polyvinylidene Fluoride Cantilevers for Synchronously Measuring Airflow Direction and Velocity, *Flow Measurement and Instrumentation* **2019**, 67, 166.

[4] S. A. Moshizi, A. Abedi, M. Sanaeepur, C. J. Pastras, Z. J. Han, S. Wu, M. Asadnia, Polymeric Piezoresistive Airflow Sensor to Monitor Respiratory Patterns, *Journal of The Royal Society Interface* **2021**, 18, 20210753.

[5] S. A. Moshizi, A. Abedi, C. J. Pastras, S. Peng, S. Wu, M. Sanaeepur, M. Asadnia, Carbon Nanofiber-reinforced Pt Thin Film-based Airflow Sensor for Respiratory Monitoring, *Sensors and Actuators A: Physical* **2022**, 347, 113969.

[6] S. Zhang, Z. Shi, J. Yuan, X. Gao, W. Cai, Y. Jiang, Y. Liu, Y. Wang, Membrane Light-Emitting Diode Flow Sensor, *Advanced Materials Technologies* **2018**, 3, 1700285.

[7] X. Tong, B. Hao, Z. Chen, H. Liu, C. Xuan, Thermal Airflow Sensor Design and Temperature Compensation Research based on the Thermostatic Method, *Sensor Review* **2022**, 42, 568.

[8] A. Abdulhameed, I. A. Halin, M. N. Mohtar, M. N. Hamidon, Airflow-assisted Dielectrophoresis to Reduce the Resistance Mismatch in Carbon Nanotube-based Temperature Sensors, *RSC Advances* **2021**, 11, 39311.

[9] Y. Liu, L. Zhao, R. Avila, et al, Epidermal Electronics for Respiration Monitoring via Thermo-sensitive Measuring, *Materials Today Physics* **2020**, 13, 100199.
